# Piezochromic Behavior of 2,4,6‐Triphenylpyrylium Tetrachloroferrate

**DOI:** 10.1002/smsc.202400106

**Published:** 2024-04-26

**Authors:** Princess Canasa, David King, Petrika Cifligu, Adrian F. Lua Sanchez, Si L. Chen, Haesook Han, Trimaan Malik, Brant Billinghurst, Jianbao Zhao, Changyong Park, George R. Rossman, Michael Pravica, Pradip K. Bhowmik, Egor Evlyukhin

**Affiliations:** ^1^ Department of Physics and Astronomy University of Nevada Las Vegas Las Vegas NV 89154 USA; ^2^ Department of Chemistry and Biochemistry University of Nevada Las Vegas Las Vegas NV 89154 USA; ^3^ Canadian Light Source, Inc. University of Saskatchewan Saskatoon Saskatchewan S7N 2V3 Canada; ^4^ High Pressure Collaborative Access Team (HPCAT) X‐Ray Science Division Argonne National Laboratory Lemont IL 60439 USA; ^5^ Division of Geological and Planetary Sciences California Institute of Technology Pasadena, CA 91125 USA

**Keywords:** diamond anvil cells, high pressures, piezochromic, pyrylium salts

## Abstract

In advanced photonics, there is a growing interest in piezochromic luminescent materials that exhibit multicolor switching, driven by their potential applications in optical recording, memory, and sensors. Here, the piezochromic behavior of 2,4,6‐triphenylpyrylium tetrachloroferrate (Py‐FeCl_4_) under high pressures from 0 to 9 GPa is reported. The observed multicolor changing properties of Py‐FeCl_4_ (yellow–orange–red–maroon–black) are found to be fully reversible upon decompression to ambient conditions. The mechanism of Py‐FeCl_4_ piezochromism is investigated via Raman, infrared, and UV–vis spectroscopy combined with powder X‐ray Diffraction. The absence of structural phase transitions as well as the abrupt shifts of bandgap values together with characteristic Raman and IR peaks within 0‐9 GPa suggests that the Py‐FeCl_4_ multicoloring switching behavior is driven by an electron transfer between the inorganic FeCl_4_^−^ anion and the organic pyrylium cation. The obtained results demonstrate that Py‐FeCl_4_ dye is a good candidate for developing high‐pressure sensing technologies designed to function in extreme environments. Moreover, due to the inherent role of molecular‐structure relationships in the pyrylium salt's photophysical properties, findings suggest the potential discovery of piezochromic behavior in other pyrylium compounds.

## Graphical abstract

Investigation of 2,4,6‐triphenylpyrylium tetrachloroferrate (Py‐FeCl_4_) at high pressure reveals its piezochromic properties. Reversible multicolor‐switching behavior of Py‐FeCl_4_ (yellow–orange–red–maroon–black) is detected in the 0–9 GPa pressure range. Raman, Infrared, and UV–vis spectroscopies with X‐ray diffraction technique suggest an electron transfer between inorganic–organic ions as the main mechanism of Py‐FeCl_4_ peizochromism.

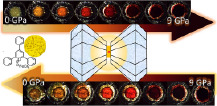

## 1 Introduction

Pyrylium salts represent a class of small organic molecules built on a six‐membered cationic heterocyclic structure with one positively charged oxygen ion and a variety of counterions, such as Cl^−^, ClO_4_^−^, BF_4_^−^, PF_6_^−^, OTs^−^, N(Tf_2_)^−^, and FeCl_4_^−^.^[^ ^1^ ^]^ As they have been investigated for more than one century, these compounds have versatile applications ranging from synthetic utility for producing heterocyclic compounds, complex macrocycles or metallo‐supramolecules, to photopolymerization reactions, photosensitizers, lasers in various organic solvents, and nonlinear optical properties.^[^ ^1^, ^2^, ^3^, ^4^ ^]^ Despite years of study and a wide range of applications, the piezochromic properties of pyrylium salts, to the best of our knowledge, have never been previously reported.

Piezochromic materials have attracted much attention because of their multicolor‐switching behavior and their applications in the field of advanced photonics, such as optical recording, memory, and sensors.^[^ ^5^, ^6^, ^7^, ^8^ ^]^ Generally, under pressure the interatomic distances within the material decrease, altering its electronic structure and subsequently shifting the bandgap energy.^[^ ^9^ ^]^ This shift causes changes in the material's optical properties, leading to observable color variations. For instance, certain materials may change color from transparent to opaque or from one color to another under pressure, a phenomenon known as piezochromism.^[^ ^10^ ^]^ The ability of a material to exhibit piezochromic properties is due to a variety of mechanisms such as a change in molecular aggregated state,^[^ ^11^ ^]^ molecular conformation,^[^ ^12^ ^]^ or alteration of chemical bonds^[^ ^13^ ^]^ with applied high pressure (HP). As a result, piezochromic materials isomerize,^[^ ^14^ ^]^ undergo structural phase changes, and^[^ ^15^ ^]^ experience lattice defects^[^ ^16^ ^]^ and modifications in electronic energy levels,^[^ ^17^ ^]^ affecting peak shape and position of their electronic absorption and emission spectra. Understanding and harnessing this behavior has significant implications for various fields, including sensor technology, where pressure‐sensitive materials can be employed to detect and visualize mechanical stress or pressure gradients.^[^ ^18^, ^19^ ^]^ Additionally, piezochromic materials hold promise for applications such as smart coatings and adaptive optics, where reversible color changes can indicate mechanical deformation or provide visual feedback in response to varying pressure conditions.^[^ ^20^, ^21^ ^]^ Although many HP studies have reported piezochromic mechanisms of synthesized novel structural phases with new optical properties, knowledge of piezochromic materials lacks universality. Moreover, in most cases, multicolor‐switching materials show full reversibility upon hydrostatic pressure release. Therefore, HP synthesis of materials stable at ambient conditions with novel structural and optical properties remains a challenge.^[^ ^5^ ^]^

In this study, we demonstrate that when 2,4,6‐triphenylpyrylium tetrachloroferrate (Py‐FeCl_4_) is subjected to HP, particularly in the 0–9 GPa pressure range, its multicolor switching behavior is observed. Furthermore, this color change evolution is completely reversible upon pressure decrease. When the samples are recovered, they exhibit similar chemical/structural properties as the material prior to compression. To unveil the main mechanism responsible for Py‐FeCl_4_ piezochromic behavior, Raman, far‐infrared (Far‐IR), and UV–vis spectroscopies combined with powder X‐ray diffraction (pXRD) were performed during a compression/decompression cycle. Our experimental findings suggest that HP‐induced electron transfer between the pyrylium cation and FeCl_4_^−^ anion is the main mechanism of Py‐FeCl_4_ color‐switching behavior. The observed piezochromism of Py‐FeCl_4_, reported in this work, indicates that this organic–inorganic salt can be utilized for designing HP sensing technologies with the ability to detect pressure changes in extreme environments. Moreover, due to a general contribution of the molecular‐structure relationship in the photophysical properties of pyrylium salts, our results suggest that piezochromic behavior could potentially be detected in various types of pyrylium dyes, thus providing a vast repertoire of novel compounds with high technological interest.

## 2 Results and Discussion

**Figure 1:**
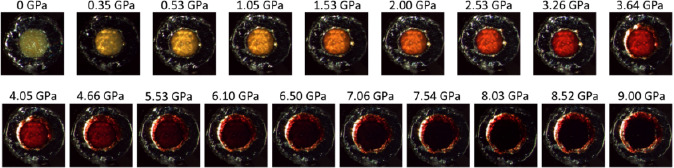
Compression cycle of 2,4,6‐triphenylpyrylium tetrachloroferrate from 0 to 9 GPa pressure range.

We have recently developed a novel means of pyrylium salts synthesis which is much safer and less expensive than previous conventional synthetic methods and does not require the use of acids as condensing agents.^[^ ^4^, ^22^, ^23^ ^]^ The schematic representation of the multistep synthesis of Py‐FeCl_4_ is presented in Figure S1, Supporting Information. The synthesized bright yellow Py‐FeCl_4_ powder was loaded into a symmetric diamond anvil cell (DAC) and was pressurized from 0 to 9 GPa with 0.5 GPa increments. **Figure** **1** shows progressive color switching behavior of Py‐FeCl_4_ with pressure increase. From ambient up to 2 GPa, a gradual change of color from yellow to orange is observed. There is increase of pressure up to 7 GPa‐induced color transformation to red, which eventually becomes maroon. In the pressure range between 7 and 9 GPa, the sample turns and remains black. During decompression, the gradual return of Py‐FeCl_4_ color is detected (see Figure S2, Supporting Information), suggesting its piezochromism is reversible. We note that a slight color difference between as‐loaded sample (0 GPa in Figure 1) and the decompressed sample to 0 GPa (see Figure S2, Supporting Information) could be due to the residual strains after the compression–decompression cycle.

**Figure 2:**
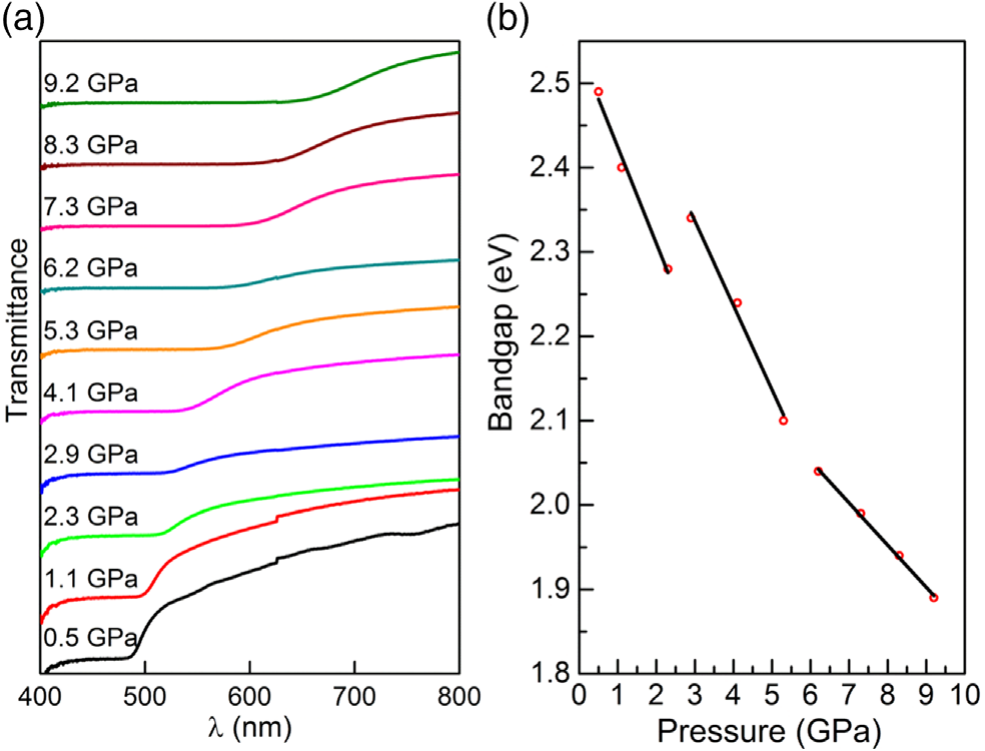
a) Transmittance spectra of Py‐FeCl_4_ from 0 to 9 GPa during the compression cycle; b) bandgap energy variations with pressure from 0 to 9 GPa pressurization.

To quantify HP‐induced Py‐FeCl_4_ multicolor behavior, its optical properties were investigated by means of UV–vis spectroscopy. Indeed, the optical bandgap energy is a critical parameter that defines the material's operational range in optical and electrical applications.^[^ ^24^, ^25^ ^]^ Under HP, the bandgap of materials may undergo significant shifts, exhibiting changes in its electronic properties.^[^ ^9^ ^]^ Typically, as pressure increases, the interatomic distances decrease, leading to alterations in the electronic structure.^[^ ^10^ ^]^ In semiconductors, for instance, this phenomenon can result in a narrowing or widening of the bandgap, influencing the material's conductivity and optical properties.^[^ ^26^, ^27^ ^]^ For instance, materials with wider bandgaps have higher‐energy photons, allowing them to absorb and emit light of shorter wavelengths, which makes them suitable for applications such as the light‐emmiting diodes and solar cells.^[^ ^28^ ^]^ In contrast, materials with narrower bandgaps are more efficient at absorbing and emitting longer‐wavelength light, finding use in technologies like photodetectors and infrared sensors.^[^ ^29^ ^]^ Thus, understanding and manipulating the optical bandgap is essential for tailoring materials to specific technological needs. Polarized optical transmission spectra in the 400–800 nm range were obtained in situ for Py‐FeCl_4_ loaded in a DAC within the 0.5–9 GPa pressure range with 1 GPa increments (see **Figure** **2**a). Upon compression, the sample transmission edge located at ≈498 nm at 0.5 GPa undergoes a shift toward higher wavelengths. At 9 GPa, it reaches the value of ≈656 nm. The bandgap energy (*E*_g_) has been calculated using the Tauc relation (See Supporting Information)^[^ ^30^, ^31^ ^]^ The obtained direct optical bandgap energy of Py‐FeCl_4_ pressurized in the 0.5–9 GPa pressure range changes from 2.49 to 1.89 eV (see Table S1, Supporting Information). Moreover, as shown in Figure 2b, during pressurization within 0–9 GPa, several regions between 0.5 and 2.3 GPa, 2.9 and 5.3 GPa, and 6.2 and 9.2 GPa with continuous bandgap change are detected; however, these regions themselves exhibit discrete behavior.^[^ ^32^ ^]^ We note that the continuous change of bandgap values might be associated with gradual changes in material properties such as temperature changes^[^ ^33^ ^]^ or compositional changes.^[^ ^34^ ^]^ On the other hand, abrupt bandgap changes could represent sudden alterations in the materials structure, electronic configuration, etc.; for instance, phase transitions,^[^ ^35^ ^]^ stoichiometry induced,^[^ ^36^ ^]^ or external factors (e.g., light or electrical stimuli)^[^ ^37^, ^38^ ^]^ could cause abrupt shifts in the bandgap. As shown in Figure 2b, in the HP range between 2.3 and 2.9 GPa, the bandgap increase is detected (see Figure 2b). One possible explanation for this anomalous behavior could involve the intricate changes in the coordination environment around the iron (Fe) ion and the chloride (Cl) ions within the molecular structure under HP. At lower pressures (below 2.3 GPa), the initial compression may lead to a decrease in bandgap due to the narrowing of electronic bands resulting from reduced interatomic distances.^[^ ^39^ ^]^ However, at 2.9 GPa, the structural rearrangement induced by compression could trigger significant modifications in the electronic configuration and bonding patterns within the pyrylium molecule.^[^ ^40^ ^]^ This could include the formation of new chemical bonds or the alteration of existing ones, potentially resulting in an increase in the bandgap. Moreover, pressure‐induced electron transfer processes between the pyrylium ring and the FeCl_4_^−^ moiety may contribute to the observed bandgap increase.^[^ ^41^ ^]^ The emergence of new electronic states or the stabilization of particular electronic configurations under extreme compression could lead to a widening of the bandgap.^[^ ^39^ ^]^

**Figure 3:**
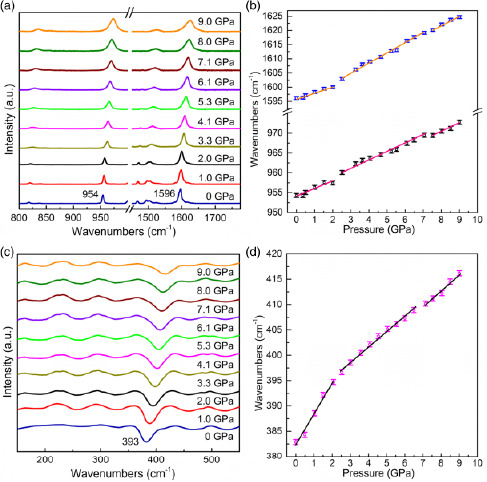
Comparison of a) Raman spectra; b) analysis of observed characteristic peak shifts; c) far‐IR spectra; d) analysis of observed characteristic peak during compression cycle from 0 to 9 GPa.

For elucidating the main mechanism of Py‐FeCl_4_ color‐switching behavior, Raman and Far‐IR spectroscopies were carried out. These spectroscopic techniques are powerful tools for investigating structural and chemical transformations in organic/inorganic materials induced by temperature, pressure, doping, etc.^[^ ^38^, ^42^, ^43^, ^44^, ^45^ ^]^ **Figure** **3** displays Raman spectra (800–1800 cm^−1^) as well as Far‐IR spectra of Py‐FeCl_4_ at selected pressure points. The complete Py‐FeCl_4_ Raman spectrum at ambient conditions is presented in Figure S4, Supporting Information. Within Py‐FeCl_4_ Raman spectrum, four characteristic peaks can be recognized: the one at ≈335 cm^−1^ corresponds to the Fe–Cl stretching motion of FeCl_4_^−^ anion,^[^ ^46^ ^]^ peaks around 950 and ≈1000 cm^−1^ (aromatic ring breathing), as well as a peak at ≈1590 cm^−1^ (benzene ring stretching) represents vibrational modes of 2,4,6‐triphenylpyrylium cation.^[^ ^47^ ^]^ It should be noted that because two of the characteristic peaks located at ≈335 and ≈1000 cm^−1^ are hindered by nearby spectral features (≈300 and ≈1015 cm^−1^), their HP behaviors could not be explicitly examined. Therefore, only two other peaks at 954 and 1596 cm^−1^, respectively, are reported here for the spectral analysis of Py‐FeCl_4_ chemical and structural transformations responsible for its piezochromic behavior within 0–9 GPa pressure range (see Figure 3a,b). Moreover, for investigating the HP evolution of vibrational modes of the FeCl_4_^−^ anion and to assess their contribution toward Py‐FeCl_4_ piezochromic behavior, the peak at 393 cm^−1^ (Fe‐Cl stretching), in the far‐IR range,^[^ ^48^ ^]^ was studied (see Figure 3c,d). As depicted in Figure 3b,d, all peaks demonstrate similar pressure‐dependent wavenumber shifts. Namely, two abrupt shifts between 2 and 3 GPa and 7 and 8 GPa pressure ranges are observed. This spectroscopic behavior corresponds to the color switching evolution seen in Figure 1, as well as with the bandgap redshift displayed in Figure 2b. Upon decompression, all peaks within the Raman and Far‐IR spectra return to their original positions, demonstrating full reversibility of Py‐FeCl_4_ chemical/structural transformations (See Figure S5–S7, Supporting Information). We note that the abrupt shifts of the characteristic peaks observed in Raman and IR spectroscopies of ≈5 cm^−1^ could be associated with instrumental error due to the limited resolution of both techniques. However, the consistency of Raman and IR spectral behaviors, together with bandgap changes within the 0–9 GPa pressure range, suggests that the abrupt shift of characteristic spectral peaks upon compression can be an indication of either structural phase^[^ ^49^, ^50^ ^]^ or electronic structure transitions.^[^ ^51^, ^52^ ^]^

**Figure 4:**
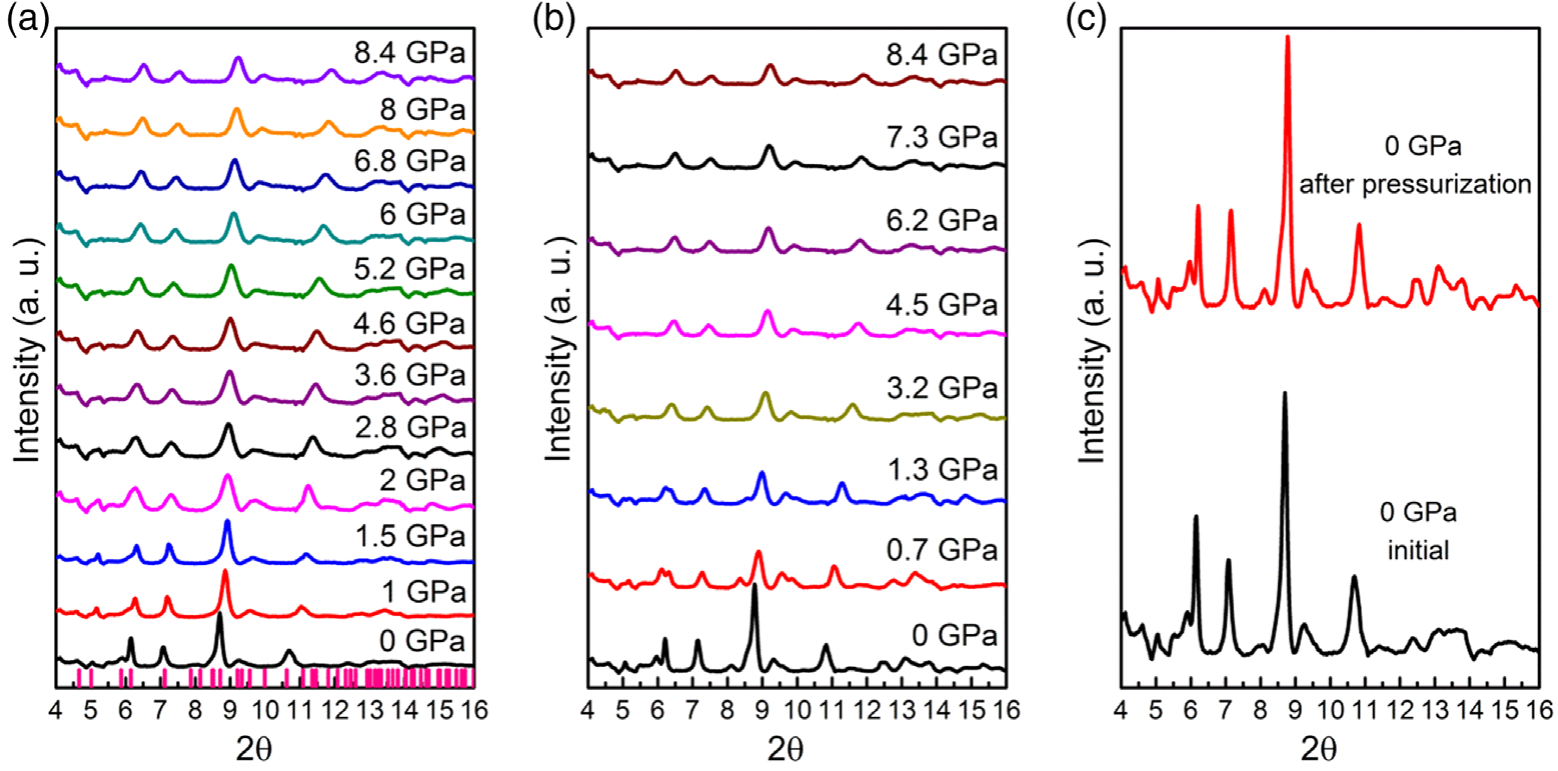
XRD patterns of pyrylium FeCl_4_: a) during the compression cycle; vertical bars indicate peak positions of previously reported orthorhombic crystal structure of pyrylium FeCl_4_; b) during the decompression step and (c) comparison of XRD spectra before and after the compression–decompression cycle from 0 to 9 GPa.

Therefore, to obtain more insights about the Py‐FeCl_4_ piezochromic mechanism, additional pXRD measurements at HP were conducted. Powdered Py‐FeCl_4_ was loaded into DACs and XRD patterns at HP were collected via monochromatic X‐rays at 16.5 keV at the 16 BM‐D beamline of the High Pressure Collaborative Access Team (HP‐CAT) at the Advanced Photon Source (APS).^[^ ^53^ ^]^ **Figure** **4**a shows XRD patterns of Py‐FeCl_4_ at selected pressures within the 0–9 GPa range. The Py‐FeCl_4_ XRD pattern at ambient pressure perfectly corresponds to the previously reported orthorhombic crystal structure of Py‐FeCl_4_ with the *Pbcm* space group and the following lattice parameters: *a* = 10.949 Å, *b* = 17.190 Å, *c* = 12.099 Å, and *β* = 90° (pink vertical bars in Figure 4a).^[^ ^54^ ^]^ Upon compression, all XRD peaks undergo a gradual shift toward higher 2Θ, indicating a reduction of the unit cell volume. However, no formations of new XRD peaks in the 0–9 GPa range are detected, demonstrating the absence of any Py‐FeCl_4_ structural transformations (see Figure 4a). We note that the detected shifts in XRD peaks could indicate that while the material maintains its initial crystalline phase, the molecules of Py‐FeCl_4_ undergo structural adjustments to accommodate the external stress (see Table S2, Supporting Information). These adjustments may include subtle modifications in molecular conformation^[^ ^40^ ^]^ or packing arrangements^[^ ^10^ ^]^ within the crystal lattice. Such alterations can lead to variations in the electronic structure,^[^ ^39^ ^]^ intermolecular interactions,^[^ ^55^ ^]^ and excitonic coupling within the material,^[^ ^56^ ^]^ influencing its optical properties and resulting in the observed piezochromic behavior. Therefore, although no phase transition occurs in the 0–9 GPa pressure range upon the compression, the observed shifts in XRD peaks suggest a dynamic response of the molecular structure to applied pressure, demonstrating the possible affection of molecular conformation, or packing pattern to the multicolor behavior of Py‐FeCl_4_. Moreover, the abrupt shift of bandgap values displayed in Figure 2b could be associated with sudden variations of the distance between FCl_4_^−^ anion and pyrylium cation at HP conditions (see Table S2, Supporting Information). During decompression from 9 GPa to ambient pressure, Py‐FeCl_4_ peaks gradually return to their original positions, as depicted in Figure 4b. Furthermore, XRD patterns of Py‐FeCl_4_ prior and postcompression are practically identical (see Figure 4c), showing the complete structural reversibility of Py‐FeCl_4_ within the 0–9 GPa pressure range. Therefore, it can be suggested that the main mechanism of Py‐FeCl_4_ piezochromic behavior is more likely related to HP‐induced electron transfer between organic–inorganic components.^[^ ^57^, ^58^ ^]^

The piezochromic process of Py‐FeCl_4_ can be proposed as follows. Under high external pressure, the molecules in the lattice are forced to move closer.^[^ ^59^ ^]^ The intermolecular contacts between the electron‐rich FeCl_4_^−^ and the electron‐deficient pyrylium moiety are shortened to favorable distances to induce electron transfer from FeCl_4_^−^ to the pyrylium ion. This leads to a metastable state with two radical sites per molecule, one delocalized over the pyrylium moiety and the other over the FeCl_4_^−^ groups. There could be intermolecular radical–radical interactions, which, together with the planar configuration of the pyrylium moiety, should help to stabilize the radical state.

One can assume that the reverse electron transfer is slow and cannot keep pace with recovery of the intra‐ and intermolecular parameters upon decompression. Therefore, although the molecular and crystal structure is completely recovered after removal of pressure, the radical state remains in a certain proportion. The back transfer process continues upon further standing at ambient pressure to finally regenerate the original state. Electron transfer leads to a shift to lower energies of an existing ligand‐to metal charge transfer band (color change depicted in Figure 1), and the spectral Raman, IR, and bandgap shifts as well as associated color change during the compression/decompression process should be attributed to the modulation effects of pressure on intermolecular interactions and molecular conformation. The piezochromic behavior of Py‐FeCl_4_ is most likely the electron transfer between electron‐rich FeCl_4_^−^ ion to the electron‐deficient pyrylium moiety. This process needs to be further studied with the HP electron spin resonance (ESR). However, to our knowledge, HP electron transfer piezochromism for an organic compound for the first time has been reported in 2017 wherein electron transfer occurs intramolecularly between electron‐rich carboxylate ion from an extended viologen‐carboxylate zwitterion (aka as inner salt) to the electron‐deficient viologen moiety.^[^ ^57^ ^]^ The same group also reported the pressure‐induced electron transfer of a series of carboxyl group terminated extended viologen with different inorganic anions. They found that the ease of solid‐state piezochromism follows the following order: Cl^−^ > Br^−^ > I^−^/BF_4_^−^/PF_6_^−^/ClO_4_^−^. The results of these studies reveal pressure‐induced electron transfer in the solid state from the anions to the electron‐deficient viologen moiety intermolecular.^[^ ^60^ ^]^ Their very recent results of piezochromism of extended viologens with carboxy groups terminated in their para‐ and metapositions with different inorganic anions NO_3_^−^, Br^−^, and BF_4_^−^ also revealed that the main mechanism is the intermolecular electron transfer from the electron‐rich inorganic anions to the electron‐deficient viologen moieties,^[^ ^61^ ^]^ which is fundamental not only to the development of solid chromic materials but also to other fields such as electronic modulation and HP science.

The observed reversible piezochromic behavior of 2,4,6‐triphenylpyrylium tetrachloroferrate opens promising possibilities of this salt in various applications. The reversible color change under high‐pressure conditions makes it particularly suitable for pressure‐sensitive optical sensors, where precise detection of pressure variations is required. Its piezochromic phenomenon could also find applications in fields such as pressure monitoring in industrial processes, biomedical devices, and environmental sensing. Moreover, understanding the relationship between pressure and optical properties, such as the shifts of bandgap energy, is critical for designing materials with tunable optoelectronic properties. Therefore, the observed multicolor switching behavior could potentially benefit the development of pressure‐responsive materials for use in flexible electronics, smart displays, and pressure‐triggered switches.

## 3 Conclusion

In conclusion, it has been experimentally demonstrated that Py‐FeCl_4_ exhibits reversible piezochromic behavior (yellow–orange–red–maroon–black) in the 0**–**9 GPa pressure range. Raman, Far‐IR, UV–vis, and pXRD spectroscopies were utilized for investigation of HP‐induced Py‐FeCl_4_ multicolor switching properties. The obtained results indicate that the main mechanism attributed to the Py‐FeCl_4_ piezochromism is due to an electron transfer between the organic cation and inorganic anion. Finally, the novel Py‐FeCl_4_ photophysical property reported in this work suggests that pyrylium salts can be employed in developing novel HP‐sensing technologies with capabilities to operate at extreme conditions.

## 4 Experimental Section

4.1

4.1.1

##### Novel Synthesis of 2,4,6‐Triphenylpyrylium Tetrachloroferrate (Py‐FeCl_4_)

Pyrylium tosylate (I) was prepared by a two‐step procedure via the diketone method according to the procedure described in the literature.^[^ ^4^, ^22^ ^]^ It was converted to the pseudobase (II) with treatment of sodium acetate in ethanol on heating for 30 min according to the reported procedure.^[^ ^23^ ^]^ The pseudobase (II) was then converted to PyCl (III) with the treatment of concentrated HCl/ethanol on heating for 1 h, according to the literature procedure.^[^ ^23^ ^]^ Finally, PyCl (III) was converted to PyFeCl_4_ on heating to reflux in acetonitrile with FeCl_3_ instead of neat condition, in the reported procedure.^[^ ^62^ ^]^ The procedure was as follows: ferric chloride (395 mg, 1.147 mmol) and 2,4,6‐triphenylpyrylium chloride (186 mg, 1.147 mmol) were dissolved in 25 mL acetonitrile and heated to reflux for 24 h. The solvent was removed under reduced pressure and the yellow solid was washed with ether to furnish 530 mg PyFeCl_4_ (91% yield). Mp: 220–225 °C.

##### Sample Preparation of Py‐FeCl_4_ with a DAC

The synthesized bright yellow Py‐FeCl_4_ powder was loaded into a symmetric DAC to generate HPs. A 250 μm‐thick stainless steel gasket was preindented to 50 μm and laser drilled with a 100–150 μm‐wide hole in the center to confine the sample in a DAC.^[^ ^63^ ^]^ To consistently measure pressure, a ruby sphere was centered into the drilled opening and then packed with solid Py‐FeCl_4_ powder.

##### UV–vis, Raman, and Far‐Infrared Spectroscopy of Py‐FeCl_4_ in a DAC

UV–vis spectra were collected at the minerology lab at the California Institute of Technology (Caltech) using their home‐built microspectrometer system, which captured polarized optical transmission spectra in the 400–800 nm range with 1 nm resolution. The microspectrometer system consisted of a 1024‐element Si connected to a grating spectrometer system via fiber optics. The spectrometer was attached to a modified NicPlan infrared microscope with a calcite polarizer.^[^ ^64^ ^]^ Raman spectra of compressed samples at room temperature were measured in the backscattering configuration using a U1000 JY spectrometer equipped with a liquid nitrogen‐cooled charge coupled device detector (Horiba Jobin Yvon) with spectral resolution of 3 cm^−1^. A 532 nm solid‐state KPT‐crystal laser (LASOS) was used to excite the DAC samples. No pressure‐transmitting media (e.g., methanol/ethanol) was used in Raman measurements as Py‐FeCl_4_ underwent a destabilization reaction (ring opening) with these types of solvents.^[^ ^23^ ^]^ Far‐IR studies were performed on the Py‐FeCl_4_ samples at the 02B1‐1 beamline at the Canadian Light Source. The collection optics and DAC were housed in front of the Fourier‐transform system with a plexiglass enclosure. The system was continuously purged from water vapor (as measured by a humidity sensor) using positive‐pressure nitrogen blowoff gas from a nearby liquid N_2_ Dewar. Far‐IR spectra were collected using a Horizontal Microscope system. Far‐IR synchrotron radiation was redirected from the sample compartment of a Bruker IFS 125 HR spectrometer to along the working distance Schwarzchild objective which focused light onto the sample. A similar objective behind the sample collected the transmitted light and directed it to an off‐axis parabolic mirror which refocused the light into an Infrared Laboratories Ge:Cu detector. The spectrometer was equipped with a 6‐μm mylar beam splitter, and the data were collected using a scanner velocity of 40 kHz, 12.5 mm entrance aperture, and a resolution of 1 cm^−1^.

##### Powdered X‐ray Diffraction of Py‐FeCl_4_ in a DAC

A tunable Si (111) double‐crystal monochromator in pseudochannel‐cut mode was used to filter and deliver X‐rays of fixed but tunable energies. The DAC sample was irradiated for 1 h with monochromatic X‐rays at 16.5 keV energy (*λ* = 0.751 Å). The horizontal and vertical full width at half‐maximum of the X‐ray beam was 4 × 4.7 μm^2^. Angle‐dispersive X‐ray diffraction patterns were collected every minute during X‐ray irradiation using a Pilatus 1 m detector. Afterward, all diffraction patterns were integrated in 2*θ* using the Dioptas program to produce intensity versus 2*θ* plots.^[^ ^65^ ^]^ We noted that the measurements were performed at room temperature.

## Conflict of Interest

The authors declare no conflict of interest.

## Supporting information

Supplementary Material

## Data Availability

The data that support the findings of this study are available from the corresponding author upon reasonable request.
