# Supplementary material for: Piezochromic Behavior of 2,4,6‐Triphenylpyrylium Tetrachloroferrate
Source: Small Sci. 2024 Apr 26;4(8):2400106. doi: 10.1002/smsc.202400106 (PMC11935120; doi:10.1002/smsc.202400106)
Supplement: Supplementary file 1 — Supplementary Material [file SMSC-4-2400106-s001.pdf]

Supporting Information

Piezochromic Behaviour of 2,4,6-triphenylpyrylium tetrachloroferrate

Princess Canasa,[a] David King,[b] Petrika Cifligu,[a] Adrian F. Lua Sanchez,[a] Si L. Chen,[b] Haesook Han,[b] Trimaan Malik,[a] Brant Billinghurst,[c] Jianbao Zhao,[c] Changyong Park,[d] George Rossman,[e] Michael Pravica,[a] Pradip K. Bhowmik,[b] and Egor Evlyukhin[a]*

[a]Department of Physics and Astronomy, University of Nevada Las Vegas, Las Vegas, NV 89154, USA.

[b]Department of Chemistry and Biochemistry, University of Nevada Las Vegas, Las Vegas, NV 89154, USA.

[c]Canadian Light Source, Inc., University of Saskatchewan, Saskatoon, Saskatchewan S7N 2V3, Canada.

[d]High Pressure Collaborative Access Team (HPCAT), X-Ray Science Division, Argonne National Laboratory, Lemont, IL 60439, USA.

[e]Division of Geological and Planetary Sciences, California Institute of Technology, Pasadena, California 91125, USA.

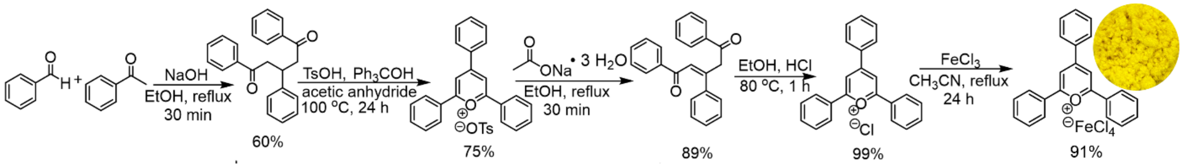

I **II** **III**

Figure S1. Multistep synthesis of 2,4,6-triphenylpyrylium tetrachloroferrate from benzaldehyde and acetophenone.

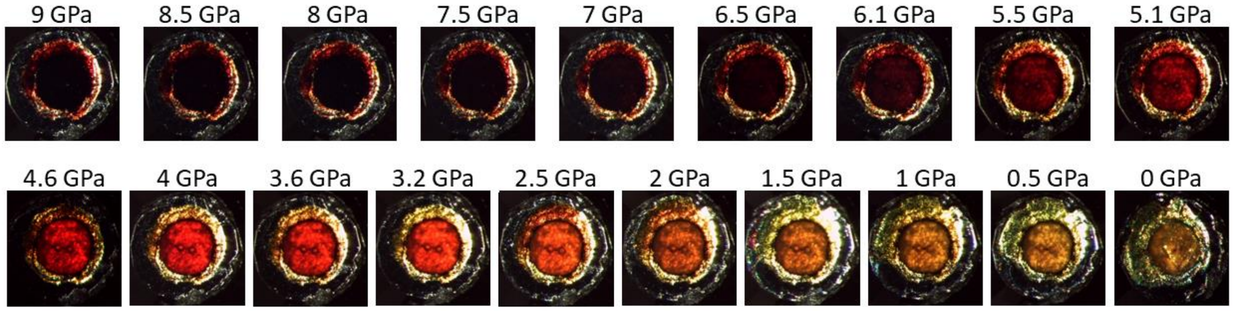

Figure S2. Decompression of 2,4,6-triphenylpyrylium tetrachloroferrate from 9 to 0 GPa.

The bandgap energy (E_g) has been calculated using the Tauc relation[1,2] $(\alpha h\nu)^n = A(h\nu - E_g)$, where A is the edge parameter, h is Planck's constant, $h\nu$ is the photon energy, α is the absorption coefficient and n is either 2 for direct band transitions or 1/2 for indirect band transitions.[3] The absorption coefficient (α) value is calculated using the relation[4] $\alpha = \mu^{-1} \times \ln(T^{-1})$, where μ is the sample thickness and T is the transmittance. The direct optical bandgap energies at the selected pressure points (see Figure 2b) are estimated from the Tauc plots of $(\alpha h\nu)^2$ versus photon energy $h\nu$ according to Kubelka–Munk theory.[5] An example of Tauc plot which corresponds to ambient pressure is displayed in Figure S3. The value of photon energy extrapolated to $\alpha = 0$ yields an absorption edge which corresponds to a bandgap energy, E_g.

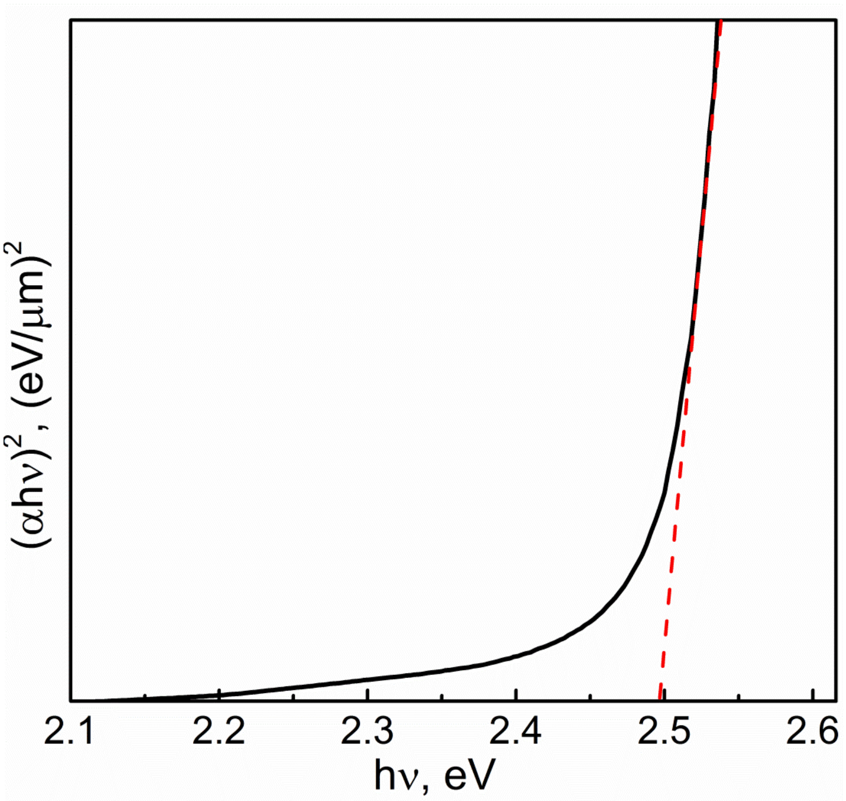

Figure S3. Variation of $(\alpha h\nu)^2$ vs. photon energy ($h\nu$) for 2,4,6-triphenylpyrylium tetrachloroferrate at ambient pressure.

Table S1. Values of pressure, thickness, and optical bandgap of 2,4,6-triphenylpyrylium tetrachloroferrate.

**Pressure, GPa**	**Thickness, μm**	**Bandgap, eV**
0.5	41	2.49
1.1	37	2.40
2.3	33	2.28
2.5	33	2.23
2.9	25	2.34
4.1	20	2.24
5.3	20	2.10
6.2	20	2.04
7.3	17	1.99
8.3	17	1.94
9.2	17	1.89

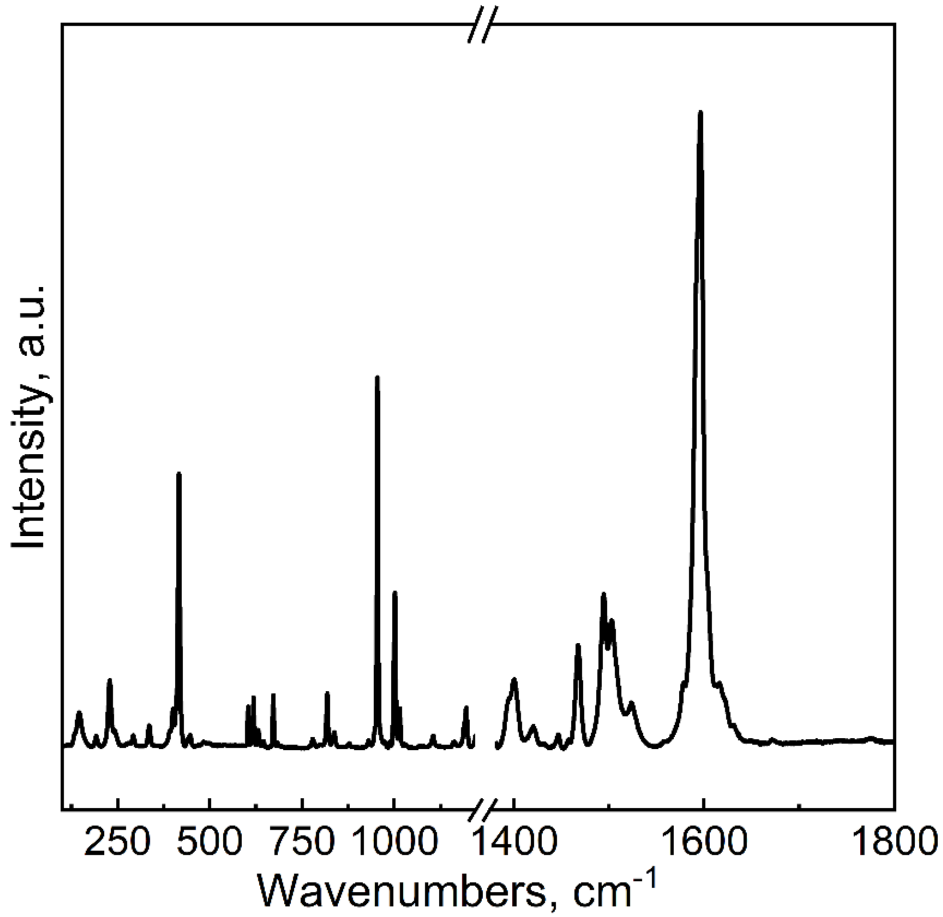

Figure S4. Raman spectrum of 2,4,6-triphenylpyrylium tetrachloroferrate at ambient pressure.

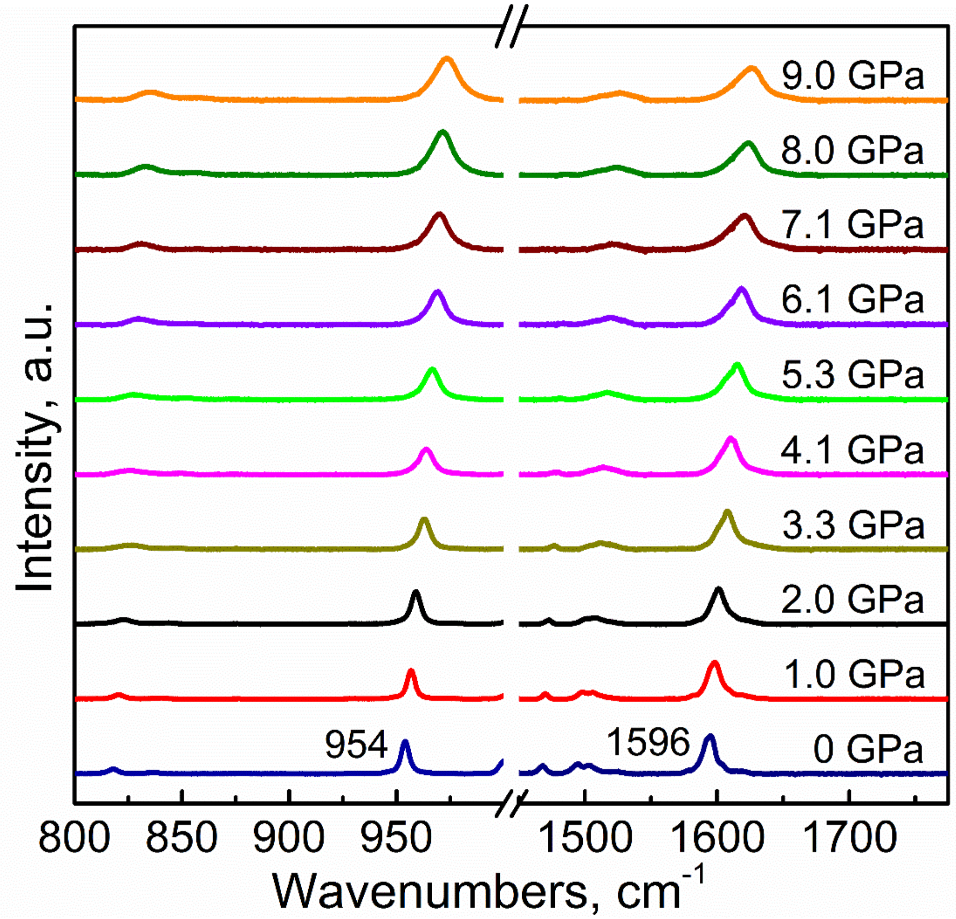

Figure S5. Raman specta of 2,4,6-triphenylpyrylium tetrachloroferrate at selected pressure points in 0-9 GPa pressure range during decompression.

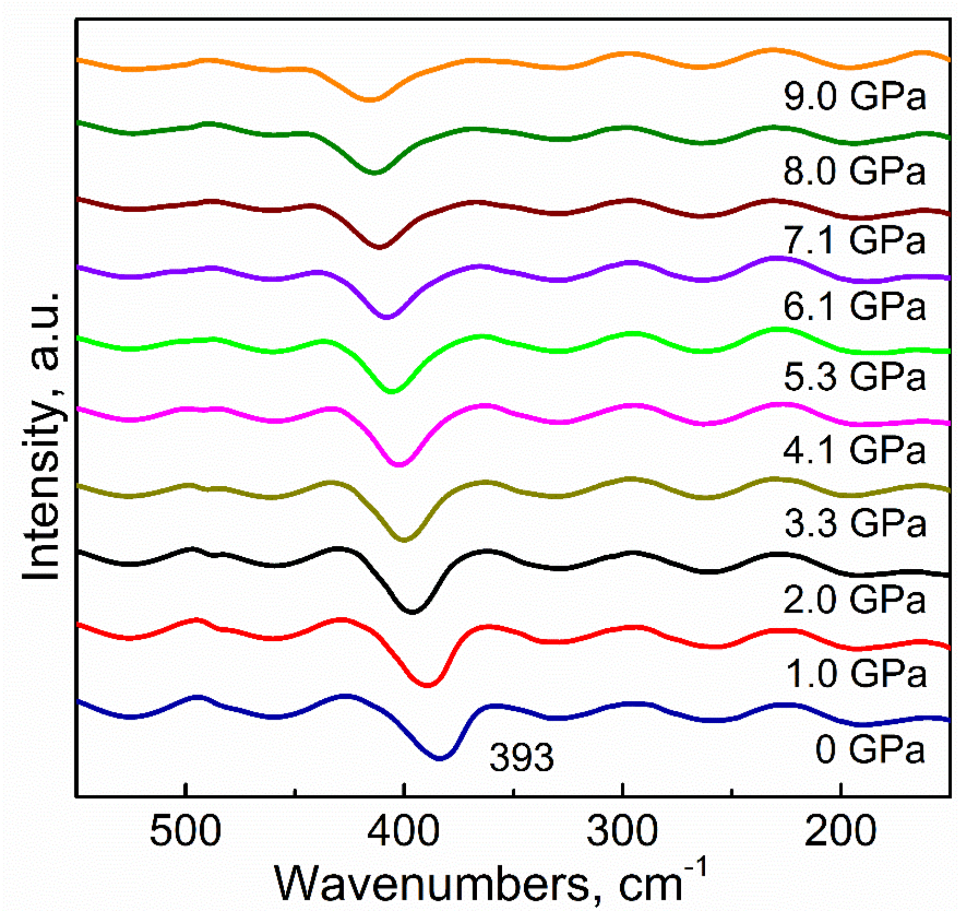

Figure. S6 Far-Infrared spectrum of 2,4,6-triphenylpyrylium tetrachloroferrate at selected pressure points in 0-9 GPa pressure range during decompression.

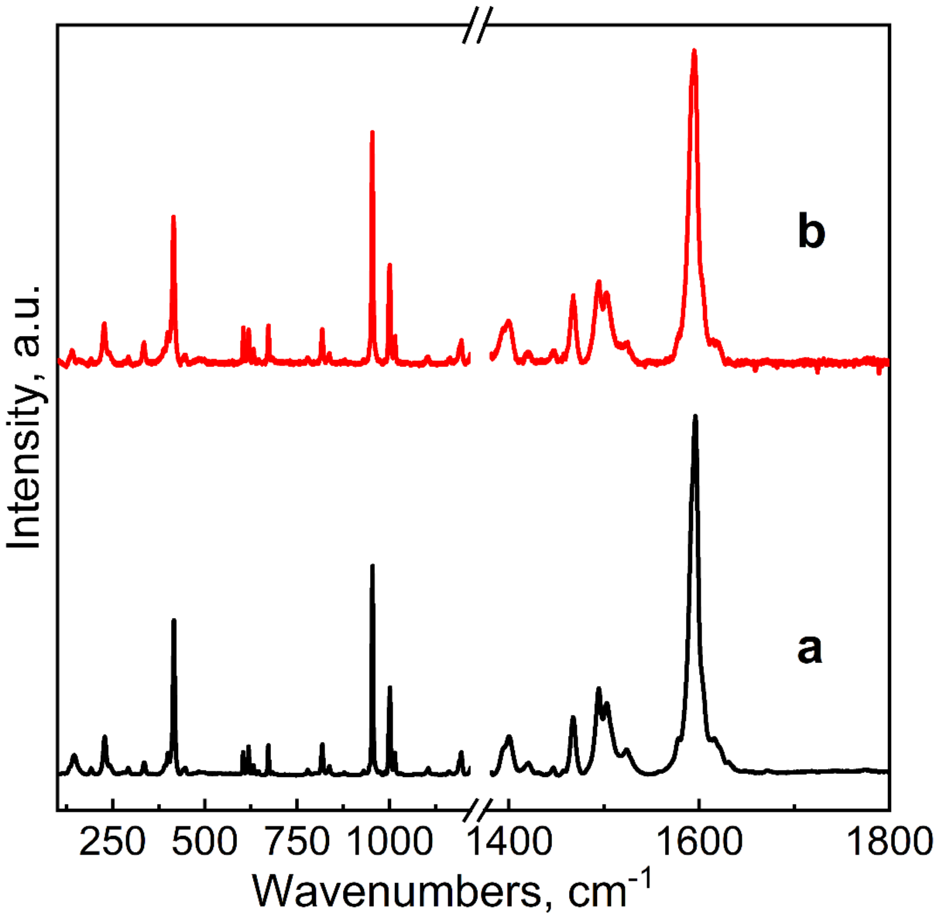

Figure. S7 Raman spectra of 2,4,6-triphenylpyrylium tetrachloroferrate at ambient pressure a) before compression and b) after decompression from 9 GPa.

Table S2. Variation of the distance between $FeCl_4$ and pyrylium cation with pressure increase. The values have been obtained from the refinement of the XRD patterns displayed in Figure 4a.

Pressure, GPa	Fe – O distance Å
0.1	5.299
1	5.20
1.5	5.164
2	5.427
2.8	5.255
3.6	5.235
4.6	5.199
5.2	5.279
6	5.705
6.8	5.638
8	5.466
8.4	5.417

References:

[1] J. Tauc, *Mater Res Bull* **1968**, *3*, 37.
[2] J. Tauc, R. Grigorovici, A. Vancu, *Phys Status Solidi B Basic Res* **1966**, *15*, 627.
[3] S. Tsunekawa, T. Fukuda, A. Kasuya, *J Appl Phys* **2000**, *87*, 1318.
[4] Triloki, R. Rai, B. K. Singh, *Nucl Instrum Methods Phys Res A* **2015**, *785*, 70.
[5] A. A. Kokhanovsky, *J Phys D Appl Phys* **2007**, *40*, 2210.
